# Changes in the Intestinal Microbiota Are Seen Following Treatment with Infliximab in Children with Crohn’s Disease

**DOI:** 10.3390/jcm9030687

**Published:** 2020-03-04

**Authors:** Kinga Kowalska-Duplaga, Przemysław Kapusta, Tomasz Gosiewski, Agnieszka Sroka-Oleksiak, Agnieszka H. Ludwig-Słomczyńska, Paweł P. Wołkow, Krzysztof Fyderek

**Affiliations:** 1 Department of Pediatrics, Gastroenterology and Nutrition, Medical College, Faculty of Medicine, Jagiellonian University, 30-633 Kraków, Poland; krzysztof.fyderek@uj.edu.pl; 2 Center for Medical Genomics—OMICRON, Medical College, Jagiellonian University, 31-034 Kraków, Poland; przemyslaw.kapusta@uj.edu.pl (P.K.); pawel.wolkow@uj.edu.pl (P.P.W.); 3 Division of Molecular Medical Microbiology, Department of Microbiology, Medical College, Faculty of Medicine, Jagiellonian University, 31-121 Kraków, Poland; tomasz.gosiewski@uj.edu.pl (T.G.); a.sroka88@gmail.com (A.S.-O.); 4 Division of Mycology, Department of Microbiology, Medical College, Faculty of Medicine, Jagiellonian University, 31-121 Kraków, Poland

**Keywords:** Crohn’s disease, inflammatory bowel diseases, microbiota, dysbiosis

## Abstract

The aim of the study was to determine the impact of biological treatment with tumor necrosis factor α antibodies (anti-TNF-α) on the intestinal microbiome of children with severe Crohn’s disease (CD) and to evaluate the differences in the intestinal microbiome between patients treated with biological therapy and healthy children. Microbiota composition was analyzed by 16S next-generation sequencing (NGS) and microbial profiles were compared between studied groups. Fifty-four samples (from 18 patients before and after anti-TNF-α induction therapy and 18 healthy children) were used in the sequencing analysis. Shannon’s diversity index (*p* = 0.003, adj. *p* = 0.010) and observed operational taxonomic units (OTUs) (*p* = 0.007, adj. *p* = 0.015) were different between controls and patients with prior therapy for CD. Statistically significant dissimilarities between beta diversity metrics, indicating distinct community composition across groups, were observed in patients with CD before and after therapy. We did not observe any differences between controls and patients with CD after therapy. Core microbiome analysis at species level showed that 32 species were present only in patients with CD but not in controls. The results show that biological treatment is associated with changes in the intestinal microbiome of patients with CD: these changes result in an intestinal microbiome pattern similar to that seen in healthy children. Long-term observation is necessary to determine whether treatment can lead to full restoration of a healthy-like microbiome.

## 1. Introduction

During the last two decades, an increase has been observed in the number of cases of inflammatory bowel diseases (IBD), particularly in the pediatric population [1,2,3]. Crohn’s disease (CD) and ulcerative colitis are two of the most important diseases belonging to the IBD group. The development of new diagnostic and therapeutic methods and intensified research efforts resulted in a better understanding of their etiology and pathogenesis, especially of the mechanisms responsible for the release, maintenance and limitation of the inflammatory process.

The results of many studies tend to indicate that a disorder of the intestinal microbiome may be one of the possible mechanisms that contribute to the pathogenesis of IBD [4,5]. Molecular sequencing of the 16S rRNA fragment enabled the identification of more than 1000 strains of bacteria in the gastrointestinal tract, while culture methods allowed the identification of only 20% of them [6,7]. Recent research points out that microorganisms, their components or excreted substances, can activate specific immune pathways depending on Th17, T regs and TR1 cells [8,9], and that diet [10,11] and genetic factors [12] can determine intestinal microbial function. Properly balanced microbiota enable and accelerate the process of mucosal healing. Studies conducted on gnotobiotic mice (germ-free mice) confirmed that the presence of commensal bacteria provides constant antigenic stimulation for abnormal chronic immune response mediated by T lymphocytes [13]. Therefore, in genetically predisposed individuals, a specific set of bacteria can exert both protective and deleterious effects. Data from studies on intestinal microbiota in IBD patients confirm the decrease in bacterial diversity and changes in specific bacterial groups abundance [4,14,15,16]. Most studies indicate the increase in various bacteria belonging to the phyla Actinobacteria and Proteobacteria and the reduction of the abundance of those belonging to the phyla Firmicutes and Bacteroidetes. Moreover, depending on the studied population of the IBD patients, changes at lower taxonomic levels were also described [4,14,17,18]. However, there are only a few studies assessing the impact of a specific treatment on the microbiome [19,20]. It seems that targeted therapeutic actions on gastrointestinal microorganisms may benefit the management of ulcerative colitis and CD patients.

Biological therapy that uses antibodies against tumor necrosis factor α (anti-TNF-α) which leads to the quelling of inflammation of the intestinal mucosa, is currently recommended and the most effective therapy for severe IBD. Infliximab (IFX), a chimeric anti-TNF-α antibody, is widely used in the treatment of pediatric patients with CD [21,22]. In addition to the undoubted benefits of biological treatment, it should be remembered that it entails a risk of adverse reactions. These include but are not limited to: acute and delayed hypersensitivity reactions, increased risk of opportunistic infections and, very rarely, the risk for malignancy such as hepato-splenic T cell lymphoma (HSTCL), reported mainly in males with concomitant exposure to thiopurines [21,23]. Several previous studies have shown that biologics contribute to the reduction of IBD-related microbial dysbiosis [24,25,26].

However, currently available data from the literature on whether biological therapy, apart from the healing of intestinal mucosa, simultaneously contributes to the normalization of microbiota in previously IFX-naïve children with CD, is limited. Therefore, the study aimed to determine what changes in intestinal microbiome occur in children with the severe forms of CD in comparison to healthy individuals and to evaluate the influence of biological treatment on the patient’s microbiome including the qualitative changes in its composition.

## 2. Materials and Methods

### 2.1. Patients

For this prospective study, we recruited anti-TNF-α naive patients with CD, aged from 2 to 18 years, who due to severe course of the disease, lack of response or developed a loss of response to previous treatment modes, were qualified to begin biological therapy. All patients were diagnosed according to the modified Porto criteria [27]. Upon inclusion into the study, disease phenotype was evaluated according to the Paris criteria [28], the intensity of endoscopic changes were assessed according to the simplified endoscopic scale for Crohn’s disease (SES-CD) [29], and disease activity was determined based on the pediatric Crohn’s disease activity index (PCDAI). In all patients, IFX (Remsima^®^, Celltrion Healthcare, Incheon, Korea) at a dose of 5 mg/kg as induction therapy was administered at 0, 2, and 6 weeks. The first fecal sample was collected before the first dose of IFX. Second assessment of disease activity (PCDAI), with assessment of endoscopic response and the analysis of the second stool sample were performed from 6 to 8 weeks after the third and last induction dose of biological treatment but before administering the next dose of the drug in maintenance therapy.

We excluded those patients who were treated with probiotics and antibiotics (during the last three months), had any confirmed infection of the gastrointestinal tract or presented with an isolated perianal fistula.

Healthy unrelated children, without IBD family members, who did not meet the exclusion criteria, were included in the control group. In this group, only one stool sample was collected.

Once obtained, the stool samples were stored refrigerated for up to 24 h, and then kept deep-frozen (−70 °C).

### 2.2. Next-Generation Sequencing (NGS) and Bioinformatic Analysis

DNA isolation from stool samples and preparation of 16S libraries were carried out according to the methodology precisely described in our previous study [30]. The obtained sequence reads were processed and analyzed using the Quantitative Insights Into Microbial Ecology 2 (QIIME2, version 2019.7) custom pipeline [31]. Briefly, the quality of demultiplexed reads was evaluated and the reads were trimmed to remove primers and poor-quality bases with cutadapt [32]. Trimmed pair-end sequences were denoised and merged with DADA2 [33] to generate amplicon sequence variants (ASVs). Next, reference-based chimera filtering was queried against the reference database (Greengenes version 13.8) at 99% similarity with vsearch [34]. Low-abundance ASVs were eliminated when they appeared in less than three samples or the number of counts across all samples was <5. SATé-enabled phylogenetic placement (SEPP) algorithm was used to build the tree for phylogenetic diversity computation [35]. QIIME2 diversity core-metrics-phylogenetic analyses were used to compute alpha and beta diversity values, and rarefaction curve analysis was used to estimate the completeness of microbial community sampling. We also computed default alpha and beta diversity metrics and generated principal coordinates analysis (PCoA) plots for each of the beta diversity metrics using Emperor [36]. Group significance between alpha and beta diversity indexes was calculated with QIIME2 plugins using the Kruskal–Wallis test and permutational multivariate analysis of variance (PERMANOVA), respectively. Generated ASVs were assigned to taxonomy using a naive Bayes classifier [37], which was pre-trained on the v3-v4 rRNA regions in Greengenes version 13.8, at 99% similarity. The bacterial composition was analyzed at class, order, family, genus and species levels. Differential abundance between groups at each taxonomic level was tested using ANOVA-like Differential Gene Expression Analysis (ALDEx2) [38]. The Benjamini–Hochberg correction for multiple testing that controls False Discovery Rate (FDR) at 0.05 was applied. The microbial dysbiosis index was calculated in R for each sample, according to Shaw et al. [5]. Briefly, the microbial dysbiosis index was defined as log_10_ of the total abundance of taxa increased in CD divided by taxa reduced in CD. For predicting KEGG (Kyoto Encyclopedia of Genes and Genomes) [39] -based functional profiles from 16S-based taxonomic profiles we use the PICRUSt (Phylogenetic Investigation of Communities by Reconstruction of Unobserved States) software v.1.1.4 [40]. For compatibility with PICRUSt we clustered our ASVs sequences with Greengenes 13.8 database, at 97% similarity, using vsearch [34] plugin in QIIME2. Statistical significance of predicted KEGG functional profiles was performed using LEfSe (Linear Discriminant Analysis Effect Size) [40] with the standard parameters (*p* < 0.05 and LDA score > 2.0).

### 2.3. Ethical Considerations

All procedures were performed in human participants were in accordance with the ethical standards of the Jagiellonian University Bioethical Committee (decision number: 122.6120.67.2015) and with the 1964 Helsinki declaration and its later amendments or comparable ethical standards. All experimental protocols were approved by the Jagiellonian University Bioethical Committee in Krakow, Poland.

The informed consent was signed by the parents or legal guardians of patients under the age of 18. Patients over the age of 16 years, in addition to the consent of their parents or legal guardians, also gave their own written consent to participate in the study.

## 3. Results

The study included 18 patients with severe CD, in whom IFX treatment was initiated, and 18 healthy children. The characteristics of the study group are presented in Table 1 with a detailed description in Supplement Material 1. At inclusion, 16 patients were treated with Mesalazin; azathioprine and methotrexate were used in 16 and 2 subjects, respectively.

During induction therapy, body weight, height, and BMI of patients increased significantly. We also observed a significant decrease of clinical disease activity (PCDAI, mean (SD): 44.75 (19.44) vs. 4.86 (4.49) points, *p* < 0.05) and of endoscopic activity (SES-CD, mean (SD): 17.22 (9.99) vs. 8.41 (7.73) points; *p* < 0.01). According to the PCDAI, all patients responded to induction therapy: 16 (88%) of them had remission (PCDAI: 0–10 points) and 2 (12%) mild disease activity (PCDAI: 10–30 points). Calprotectin concentration (mean (SD)) dropped significantly from 1920.44 µg/g (1326.73) to 629.17 µg/g (68.5.92) (*p* < 0.05). At least a threefold decrease in the calprotectin concentration compared to the baseline was observed in 12 (67%) patients. All but one patient in whom calprotectin levels decreased slightly or increased after the 3rd dose of IFX presented with moderate (*n* = 4; SES-CD: 7–15 points) or severe (*n* = 1, SES-CD > 15 points) endoscopic activity of the disease at the controlled colonoscopy.

### 3.1. 16.S rRNA Sequencing Analysis

After DADA2 denoising, and removal of chimeras and filtering, 54 samples were included in the sequencing analysis. The 54 samples provided a median 35,546 reads with IQR 25,051–43,930. The DADA2 pipeline resulted in 369 features (ASVs), with a total frequency of 1,854,442. Based on the rarefaction curve, the diversity analysis was calculated with a minimum number of 13,396 sequences per sample, which correspond to the minimum frequency per sample.

### 3.2. Diversity Analysis

**Figure 1:**
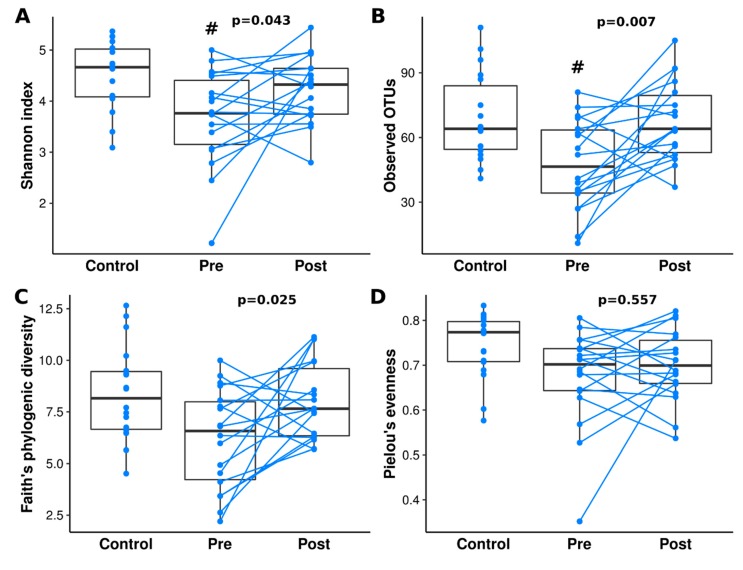
Differences in alpha diversity analysis of control and patients with CD (Crohn’s disease) pre- and post-IFX therapy. Alpha diversity was measured by the Shannon index (**A**), observed OTUs (**B**), Faith’s phylogenetic diversity (**C**), and Pielou’s measure of species evenness (**D**). Kruskal-Wallis with post-hoc was performed to analyze statistical significance. Statistically significant values, after Benjamini-Hochberg correction, between control and other groups were represented as “#”. Paired t-test *p*-values are shown above the patients with CD bars. Lines connect paired samples from the same individual.

To explore the bacterial richness and diversity, we calculated four alpha diversity indices: Shannon’s diversity index, observed OTUs, Faith’s Phylogenetic Diversity and Pielou’s evenness (Figure 1).

Only Shannon’s diversity index (*p* = 0.003, adj. *p* = 0.010) and observed OTUs (*p* = 0.007, adj. *p* = 0.015), but not Faith’s Phylogenetic Diversity (*p* = 0.025, adj. *p* = 0.074) and Pielou’s evenness (*p* = 0.027, adj. *p* = 0.080) were different between controls and patients with CD prior to therapy (CD-pre IFX). As for differences between controls and patients with CD post-therapy (CD-post IFX), we did not observe any differences in calculated alpha diversity metrics. However, we observed differences in observed OTUs between patients with CD pre- and post-therapy (*p* = 0.010, adj. *p* = 0.015). Detailed statistical results of pairwise comparisons are provided in Supplement Material 2. We also performed paired analysis of patients with CD pre- and post-therapy samples, where we observed differences in Shannon’s diversity index (M_d_ = 0.29, *p* = 0.043), observed OTUs (M_d_ = 18.5, *p* = 0.007), and Faith’s Phylogenetic Diversity (M_d_ = 1.94, *p* = 0.025), but not in Pielou’s evenness (M_d_ = 0.01, *p* = 0.557).

**Figure 2:**
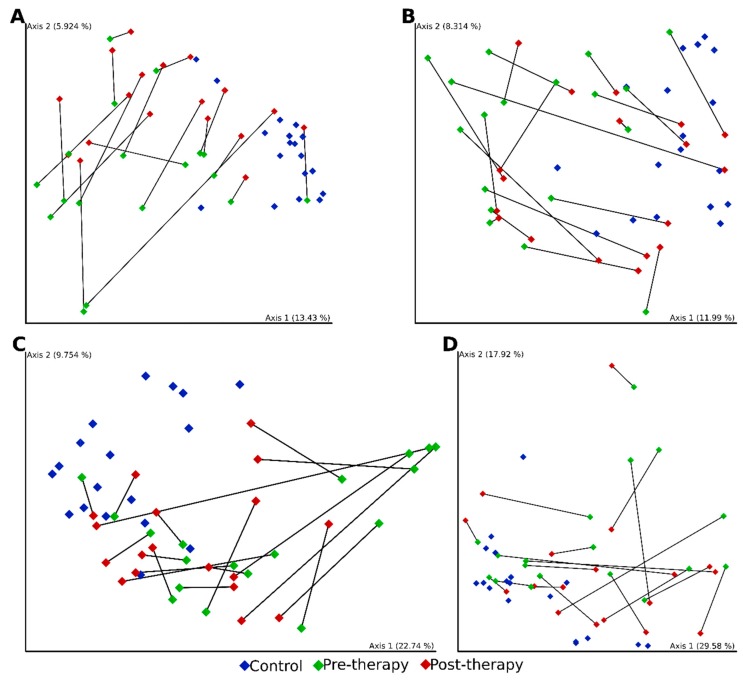
PCoA 2D plots of beta diversity analysis of control and patients with CD pre- and post-IFX therapy. Beta diversity was measured by Jaccard distances (**A**), Bray–Curtis distance (**B**), unweighted UniFrac distances (**C**) and weighted UniFrac distances (**D**). Colors indicate groups: control—blue; patients with CD pre-IFX therapy—green; patients with CD post-IFX therapy—red. Lines connect paired samples from the same individual.

Additionally, to explore the diversity of the microbial community between the groups, we calculated four beta diversity indices: Jaccard distance, Bray–Curtis dissimilarity, unweighted and weighted UniFrac (Figure 2).

Statistically significant dissimilarities between beta diversity metrics, indicating distinct community composition across groups, were seen in every pairwise comparison, except weighted UniFrac between CD patient pre- and post-therapy (*p* = 0.121, adj. *p* = 0.121). All statistical results with *p*-values and adjusted *p*-values from pairwise comparisons are shown in Supplement Material 3. Principal coordinates analysis of beta diversity showed a grouping pattern, identified in most of the subjects in every group. After induction biological therapy we observed trend in the microbial change, however, there were still differences compared to a healthy microbiome.

### 3.3. Bacterial Profile

**Figure 3:**
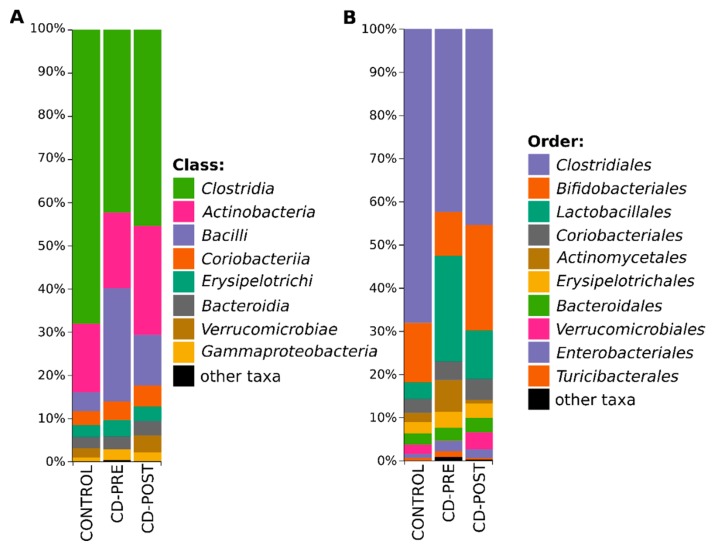
Relative abundance of most common bacterial classes (**A**) and orders (**B**) at each group. All taxa with abundance below 1% are represented as ‘other taxa’.

After annotation of 369 discovered ASVs with Greengenes taxonomy, we were able to classify 100% ASVs at the phylum (L2), 99.8% at class (L3), 99.1% at order (L4), 94.5% at family (L5), 72.9% genus (L6), and 26.6% at species (L7) level. The taxonomy-based analysis showed that the investigated microbial community consists of 7 phyla, 13 classes, 19 orders, 46 families, 96 genera and 146 species. Firmicutes (75.14% vs. 72.39% vs. 60.46%), Actinobacteria (19.17% vs. 21.81% vs. 30.13%), Bacterioidetes (2.64% vs. 3.00% vs. 3.40%), Verrucomicrobia (2.13% vs. 0.02% vs. 3.90%) and Proteobacteria (0.89% vs. 2.73% vs. 2.05%) were the most abundant phyla across all subjects (control vs. patients with CD, CD-pre IFX vs. CD-post IFX). Other identified phyla, such as TM7, and Tenericutes had a relatively low abundance (<1%). The most common taxa at class and order levels clearly showed a change in microbiome composition between control and patients with CD (Figure 3).

**Figure 4:**
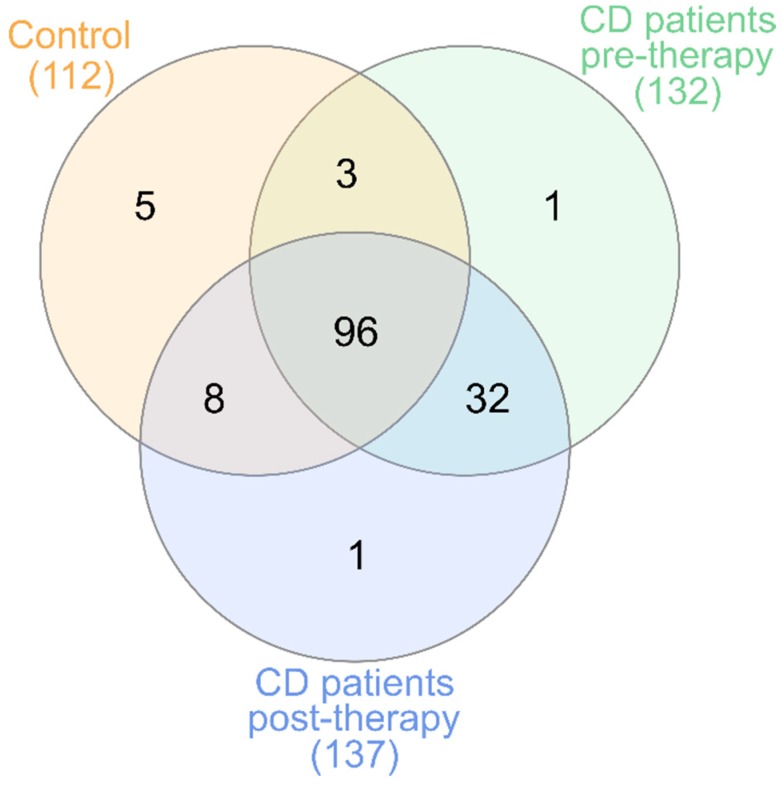
Analysis of species (L7) overlaps between different groups. Taxonomy was assigned with a 99% similarity with the Greengenes 13.8 database. Additional information about overlaps is presented in Supplement Materials 4 and 5.

Additional core microbiome analysis at species level showed that 96 species were shared between healthy patients and patients with CD regardless of the time point, although patients with CD had an addition to core microbiome composed of 32 species that were still present after therapy, and were not observed in healthy children (Figure 4). Detailed taxonomic information about overlaps between groups is available in Supplement Material 4. Short annotation of these 32 bacteria, observed only in patients with CD, in the context of human health is presented in Supplement Material 5.

### 3.4. Gut Microbiome Differences and Dysbiosis between Groups

**Figure 5:**
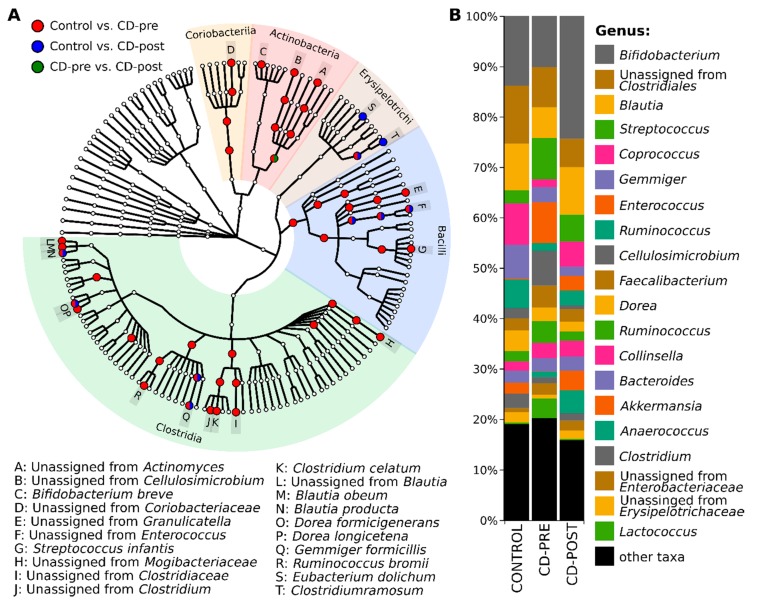
Taxa that differ significantly between control and patients with CD pre- and post-IFX therapy (**A**), and a bar plot representing the relative abundance of top 20 genera (**B**).

To better understand the changes in the microbial community during the therapy, we performed pairwise differential abundance comparison with ALDEx2, on the order through species levels. In total 49 taxa (1 class, 4 orders, 10 families, 14 genera, and 20 species) had significantly different abundance (adj. *p* value < 0.05) between the groups (Figure 5A). Detailed information from pairwise comparisons with ALDEx2 on all taxonomical levels is shown in Supplement Material 6. The differential abundance results of the three groups did not overlap substantially, the most differences (47 taxa) were observed between controls and CD-pre IFX group. Regardless of the relative abundance in the CD-post IFX group, it was closer to the controls than the CD-pre IFX group (Figure 5B). There were only 10 taxa with differential abundance between controls and CD-post IFX group. Interestingly, eight of those taxa did not change their abundance after IFX therapy (Supplement Material 6). Briefly, their fold changes (FC) were: family Enterococcaceae (−6.79 vs. −7.79); genera *Enterococcus* (−7.79 vs. −8.45), *Gemminger* (9.33 vs. 10.23) and *Clostridium* (−5.58 vs. −7.51); species *Blautia producta* (−6.76 vs. −6.76), unclassified species from *Enterococcus* (−6.93 vs. −8.29), *Dorea formicigenerans* (5.02 vs. 5.98) and *Gemmiger formicilis* (9.74 vs. 10.44) between control vs. CD-pre IFX and controls vs CD-post IFX, respectively. When we compared the CD pre- and CD post-IFX therapy we found only one significant taxon, order Actinomycetales, where abundance was decreased after therapy (FC = −2.84). Interestingly, the order Actinomycetales, the only one that significantly differed among patients with CD at the two-therapy time points, is not captured by dysbiosis index. The analysis of the differential abundance with ALDEx2 discovered two taxa from Actinomycetales, where relative abundance was significantly increased in CD-pre IFX in comparison to controls: unclassified species from *Actinomyces* and unclassified species from *Cellulosimicrobium*. After further exploring we noticed that the unclassified species from *Cellulosimicrobium*, was the most abundant one in the Actinomycetales order, and additional paired test on this species showed that its abundance was significantly decreased after therapy (M_d_ = 2.02%, *p* = 0.005).

**Figure 6:**
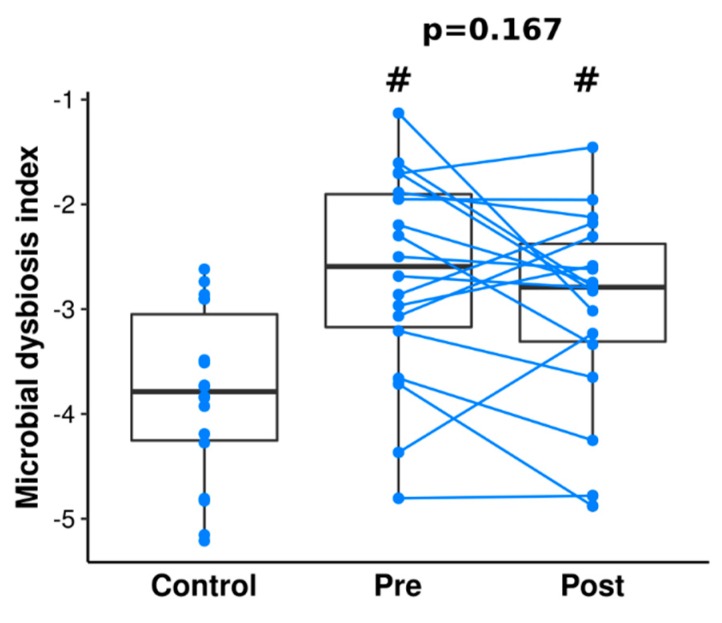
Microbial dysbiosis index of the control and patients with CD pre- and post-IFX therapy. Statistically significant values, after Benjamini-Hochberg correction, between control and other groups are represented as “#”. *p*-values from a paired t-test are shown above the bars of the patients with CD. Lines connect paired samples from the same individual.

Additionally, we calculated the dysbiosis index to determine the change in taxa associated with CD (Figure 6). While we observed differences between dysbiosis index between controls and patients with CD at pre- and post-IFX therapy (*p* = 0.003 and *p* = 0.008, respectively), we did not find changes after therapy (*p* = 0.389).

### 3.5. Functional Profiling with KEGG Pathway Functions

**Figure 7:**
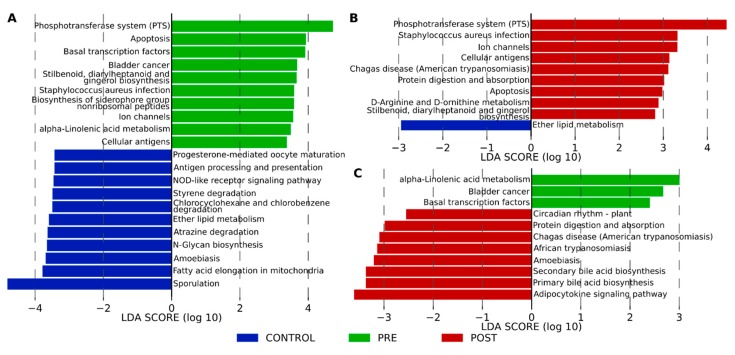
Functional divergence between the fecal microbiota of healthy controls and patients with CD pre- and post-IFX-therapy at the KEGG pathway level three. Comparison were performed between control vs CD pre-IFX-therapy (**A**), control vs CD post-IFX-therapy (**B**), and patients with CD pre- vs. post-IFX-therapy (**C**).

Using LEfSe we explored the derived KEGG pathways that were differentially expressed in the control and patients with CD pre- and post-IFX therapy. At level two we found only one pathway, the immune system, which was enriched in the controls (LDA score = 3.96, *p* = 0.049) compared to CD-pre-IFX therapy. At KEGG pathway level three we found multiple metabolic pathways differentially enriched in the control and patients with CD pre- and post-IFX therapy (Figure 7). The most different metabolic pathways were observed between controls and CD pre-IFX (Figure 7A). In the controls, they were mostly associated with metabolism (ether lipid metabolism and fatty acid elongation in mitochondria, atrazine degradation, chloroalkene and hloroalkene degradation, and styrene degradation) and immune system (antigen processing and presentation, and NOD-like receptor signaling pathway), whereas in the CD-pre-IFX therapy group they were associated with metabolism (Stilbenoid, diarylheptanoid and gingerol biosynthesis, α-Linelonic acid metabolism, and biosynthesis of siderophore group nonribosomal peptides), and environmental information processing (phosphotransferase system (PTS), cellular antigens, and ion channels). After the IFX therapy, we observed nine enriched pathways in comparison to controls, out of seven was also observed in the control compared to CD pre-IFX patients. This suggests, that even after the therapy the patients’ microbiomes could still use different metabolic pathways, compared to controls. Interestingly, we can see that the introduction of the therapy in patients with CD (Figure 7C), seems to induce pathways involved in metabolism (protein digestion and absorption, primary and secondary bile acid biosynthesis) and immune response (adipocytokine signaling pathway). Interestingly, the adipocytokine signaling pathway is a signaling cascade arising from TNF-α.

## 4. Discussion

Numerous studies have confirmed the occurrence of dysbiosis in patients with IBD. Although Firmicutes, Actinobacteria, Bacterioidetes, Verrucomicrobia, and Proteobacteria are the most abundant phyla both in health and in disease [41,42], the richness of each of these groups and diversity within them changes in disease. The authors emphasize low microbial diversity in IBD caused mainly by the loss of normal anaerobic bacteria [43,44,45].

In patients with IBD, a lower abundance of bacteria of such taxa as Bacteroidales, Erysipelotrichaceae, some Clostridiales (e.g., *Faecalibacterium prausnitzii*), *Bifidobacterium* and *Lactobacillus* is observed, while Enterobacteriaceae (e.g., *E. coli)*, Pasteurellaceae, Fusobacteriaceae, Neisseriaceae, Veillonellaceae, Gemellaceae, *Ruminococcus,* and *Clostridium* spp. are more abundant than in healthy subjects [14,20].

Andoh et al. analyzed the fecal microbiota profiles in Japanese patients with CD at different stages of disease activity and therapy. They found significantly reduced bacterial diversity in patients with CD both in the active and remission phases as compared with findings in healthy individuals [46]. Similarly, Wang et al. observed that the reduction in biodiversity and alteration in the composition of the microbiome was diminished during IFX therapy [24], and likewise, we observed dysfunction of the intestinal microbiome in pediatric patients with CD by dysregulation of multiple KEGG pathways associated with metabolism. In addition, we observed differentially enriched pathways associated with immune response. In the control and CD pre-IFX patients, they concerned the disorder of the signaling pathway of NOD-like receptors. This is of interest because mutations in NOD2 are risk factors for CD [47,48] and contributes to the shifts in the composition of the patients’ intestinal microbial community [49]. Furthermore, we observed an upregulated TNF-α signaling pathway after IFX-therapy, which may confirm the impact of treatment on metabolic activities in the fecal microbiota of patients with CD.

Longitudinal studies investigating the effects of anti-TNF treatment on gut microbiome structure in adult patients confirmed that after 30 weeks of therapy there were no significant differences in microbiome diversity in patients and healthy controls [50]. Our study confirmed the low microbial diversity in patients with CD in the active disease stage (i.e., pre-IFX therapy) when compared to controls and patients with CD after remission induction therapy. We did not observe any significant differences in calculated alpha diversity metrics between controls and patients with CD after induction therapy (Figure 1). This confirms that short-term induction IFX treatment reduces dissimilarity between patients with CD and controls, but it does not restore a typical healthy microbiome. Our analysis also revealed that there are 32 species present in patients with CD both before and after therapy, which are not observed in healthy children (Figure 4). In contrast, Mustafa et al. did not find a single species that would be universally shared among an adult IBD cohort [51]. Among these 32 species, there are both potential pathobionts and bacteria considered as probiotics. At this stage, we can only speculate that this set of species plays a role in Crohn’s disease. However, it cannot be excluded that the presence of these bacteria in patients and their absence in healthy people is a consequence of previous treatment or diet. Nevertheless, as most of those species belong to potential pathobionts, they may contribute to the maintenance of CD dysbiosis. Persistent, independent of treatment, dysbiosis was also observed in other pediatric studies [5,52].

Considering a small group of patients and the short duration of our study, we cannot draw firm conclusions. However, we believe that the continuation of this study and further monitoring of changes in the microbiome in the treated patients will allow us to determine whether these differences persist during long-term treatment.

Other authors found that three-month treatment with adalimumab (ADA; other anti-TNF-α biologics) resulted in the recovery of Firmicutes and the decrease of *Ruminococcus* and *Clostridium* spp. They concluded that the treatment with ADA leads to subtle changes in mucosal microbiota, the composition of which shows a similarity with healthy individuals [20]. We can report similar changes in IFX-treated patients as an abundance of microbiota after induction therapy was closer to control however, the treatment did not result in the acquisition of a fully healthy microbiome. We could still find a higher abundance of bacteria belonging to the family Enterococcaceae, genus *Clostridium*, and, unclassified species from Enterococcaceae in treated patients than in controls. Despite the unclear role of Enterococcaceae in IBD etiology, in our previous study, we observed a significant increase in this species in the mucosal biopsies obtained from the inflamed sites in IBD patients [16]. Since in the current study, a reduction in inflammatory changes of the mucous membrane assessed with the SES-CD scale was observed in most of our patients, it is possible that the decrease in Enterococcaceae abundance occurs only after a longer period of treatment.

Furthermore, we have shown that unclassified species from the genus *Cellulosimicrobium* belonging to the family Promicromonosporaceae was the most abundant species in this family, and also revealed a different abundance in patients before and after induction therapy. *Cellulosimicrobium* is a Gram-positive bacteria that can cause infection in susceptible individuals, including patients with CD [53]. Further research is needed to explain the relevance of this pathobiont genus in the monitoring of the process of illness or healing in patients with CD.

In the majority of studies, the low level of *Blautia, Anaerostipes, Lachnospira, Roseburia, Faecalibacterium, Coprococcus,* and *Ruminococcus* [15,54] before treatment and the increase after treatment was observed. These are SCFA (short-chain fatty acids)-producing bacteria, which are the main source of energy for colonocytes, create conditions for proper intestinal colonization and show immunomodulatory and anti-inflammatory properties. Long-term studies evaluating the response to treatment depending on the initial microbiome and its change during therapy indicate that SCFA-producing bacteria play an important role. Therefore, some authors postulate that the metabolic function rather than the taxonomic composition may contribute to the response to the treatment [50,55].

An unquestionable advantage of our work is that it concerns a homogeneous group of patients, as only patients with CD without prior biological treatment, were evaluated. The disadvantage is the small size of the study group and the relatively short observation time. Nevertheless, based on the results of our observation, we can conclude that biological treatment will change the microbial status of patients, but long-term observation is necessary to confirm this trend and its persistence as well as to determine whether treatment can lead to the restoration of the proper microbiome.

At present, it is difficult to determine whether dysbiosis in patients with IBD is a cause or consequence of the disease. Perhaps there is a vicious circle mechanism: inflammation is the cause of dysbiosis and dysbiosis increases inflammation. The existing principal methods of IBD treatment (conventional treatment and biologics) are aimed primarily at changing the pathological immune response, rather than at the causative factor, which is not fully understood. However, if the abnormal bacterial microbiota is the cause of initiating or maintaining inflammation, microbiome-modifying treatment may be an action targeted at the causative factor (or one of the causative factors). Thus, the use of appropriately selected pre- or probiotics can be a supportive treatment for existing therapies [56].

There are also speculations based on the results of preliminary studies that the initial (pre-treatment) taxonomic composition of the gut microbiome can predict the response to the immunomodulatory therapy, can be a marker of response and can help in personalizing treatment [19,25,52,57,58].

Current methods for profiling the microbiome typically utilize next-generation sequencing applications that are expensive, slow, and complex. Currently, work on the development and validation of a paper-based diagnostic platform for portable, low-cost detection of biologically relevant RNAs is ongoing with promising results [59]. In the future, this method may become a useful tool in monitoring therapeutic manipulations of the microbiome in everyday clinical practice.

The examination of the microbiome of stool is a non-invasive and non-threatening method for the patient. For pediatric patients in whom endoscopic studies, in addition to burdensome preparation, often require general anesthesia, the ability to monitor response to treatment based on the assessment of microbiome modification may be an additional prognostic factor alongside currently used clinical scales and calprotectin testing.

## Figures and Tables

**Table 1:** Baseline characteristics of the study groups.

Characteristics	Biological Therapy—IFX (*n* = 18)	Control Group (*n* = 18)
Male:Female, *n* (ratio)	11:7 (1.57)	8:10 (0.8)
Age at Diagnosis, months	147.18 ± 45.56	N/A
Age at Inclusion into the Study; months	160.28 ± 44.56 *	138.28 ± 35.16 †,*
Weight kg	44.25 ± 14.23	41.75 ± 17.37
Height, cm	152.16 ± 18.67	146.67 ± 20.52
BMI, kg/m^2^	18.54 ± 3.17	18.3 ± 3.49

† Age at sampling: mean (months); mean ± standard deviation; * *p* > 0.05. IFX—Infliximab.

## Supplementary Materials

The following are available online at https://www.mdpi.com/2077-0383/9/3/687/s1, Supplement Material 1: Table with detailed patient’s characteristics. Supplement Material 2: Differences in alpha diversity analysis of control and patients with CD pre- and post-IFX therapy. Supplement Material 3: Differences in beta diversity analysis of control and patients with CD pre- and post-IFX therapy. Supplement Material 4: Detailed taxonomic information about bacterial profile overlaps between groups and taxonomic assignment. Supplement Material 5: Short annotation of 32 bacteria observed only in patients with CD in the context of human health. Supplement Material 6: Differences in bacterial abundances between control and patients with CD pre- and post-IFX therapy discovered with ALDEx2.
