# Supplementary material for: Changes in the Intestinal Microbiota Are Seen Following Treatment with Infliximab in Children with Crohn’s Disease
Source: J Clin Med. 2020 Mar 4;9(3):687. doi: 10.3390/jcm9030687 (PMC7141282; doi:10.3390/jcm9030687)
Supplement: Supplementary file 1 [file jcm-09-00687-s001.zip › Supplement1.docx]

**Supplementary Material 1. Detailed patient's characteristics**

| **Patient ID** | **Paris**  **classification** | **Pre-IXF treatment** | | | | | | | | | **Post-IFX indution therapy** | | | | | | | | |
| --- | --- | --- | --- | --- | --- | --- | --- | --- | --- | --- | --- | --- | --- | --- | --- | --- | --- | --- | --- |
|  |  | **Weight, kg;**  **mean (SD)** | **Height, cm;**  **mean (SD)** | **BMI, kg/m^2^**  **mean (SD)** | **Total protein level (g/l)** | **Total albumin level (g/l)** | **Calprotectin (ug/g)** | **CRP** | **PCDAI** | **SES-CD** | **Weight, kg;**  **mean (SD)** | **Height, cm;**  **mean (SD)** | **BMI, kg/m^2^**  **mean (SD)** | **Total protein level (g/l)** | **Total albumin level (g/l)** | **Calprotectin (ug/g)** | **CRP** | **PCDAI** | **SES-CD** |
| 1Z1P | A1bL4aL3B2B3G0p | 32.7 | 151 | 14.34 | 69.4 | 36.4 | 1445 | 12.1 | 57.5 | 28 | 39.4 | 153 | 16.83 | 75.9 | 42.7 | 1332 | <5 | 10 | 30 |
| 2Z1P | A1bL2B1G1p | 48.35 | 155 | 20.12 | 79 | 43.5 | 1800* | 17.1 | 17.5 | 0 | 46.4 | 155 | 19.31 | 86.9 | 40.7 | 1201 | 29.5 | 10 | 14 |
| 3Z1P | A1bL2B1G1 | 38 | 154.5 | 15.92 | 68.6 | 32 | 1800* | 44.2 | 60 | 22 | 44 | 155 | 18.31 | 81.5 | 32 | 1657 | 8 | 15 | 13 |
| 5Z1P | A1bL4aL2B1G0 | 70.85 | 167.5 | 25.25 | 63.6 | 34.9 | 1800* | 14.8 | 50 | 11 | 73.8 | 167.5 | 26.3 | 72 | 37.3 | 100 | <5 | 5 | 14 |
| 9Z1P | A1bL3B1G0 | 53.4 | 174 | 17.64 | 80.8 | 43.7 | 1800* | 37.8 | 52.5 | 30 | 59.9 | 174 | 19.78 |  |  | 400 | <5 | 0 | 2 |
| 10Z1P | A1aL4aL3B1G0p | 39.45 | 143 | 19.29 | 74.2 | 45.7 | 875 | <5 | 15 | 10 | 42.75 | 143.5 | 20.76 | 73.1 | 45.3 | 100 | <5 | 15 | 0 |
| 11Z1P | A1bL4aL3B1G1 | 37.8 | 160.5 | 14.67 | 61.9 | 31.9 | 1800* | 18.6 | 55 | 21 | 44.7 | 164.5 | 16.52 | 74.6 | 45 | 100 | 4 | 0 | 9 |
| 13Z1P | A1bL4aL2B1G1 | 38.9 | 153.5 | 16.51 | 62.5 | 38.4 | 938 | <5 | 52.5 | 8 | 44.7 | 157 | 18.13 | 72.7 | 43 | 165 | <5 | 0 | 14 |
| 14Z1P | A2L3B2B3G0 | 53 | 160 | 20.7 | 82.5 | 47.9 | 90 | <5 | 0 | 0 | 54 | 161 | 20.83 | 78 | 44 | 120 | <5 | 0 | NA |
| 16Z1P | A1aL2B1G1 | 13.95 | 96.5 | 14.98 | 73 | 41 | 803 | 8.3 | 47.5 | 12 | 15.6 | 101 | 15.29 | 74.5 | 37.9 | 1800* | <5 | 5 | 6 |
| 18Z1P | A2L4aL3B2G0 | 53.7 | 168 | 19.3 | 74.3 | 42.6 | 1800* | 39.6 | 47.5 | 22 | 58.95 | 168.5 | 20.76 | 74.7 | 44.2 | 90 | <5 | 0 | 4 |
| 19Z1P | A1aL2B1G1 | 21.75 | 116 | 16.16 | 61.3 | 34.7 | 1800* | 5.5 | 65 | 9 | 22.3 | 117 | 16.29 | 77 | 43.7 | 1800* | <5 | 0 | 8 |
| 20Z1P | A1bL4aL3B1G0 | 37.5 | 153.7 | 15.87 | 76.4 | 44.1 | 1800* | <5 | 55 | 20 | 37.2 | 154 | 15.69 | 79.3 | 44.1 | 455 | <5 | 5 | 12 |
| 21 Z1P | A1bL3B1G1p | 44.95 | 149.5 | 20.11 | 67.8 | 40.1 | 621 | <5 | 10 | 11 | 50 | 149.5 | 22.37 | 70.3 | 42.7 | 100 | <5 | 5 | 0 |
| 22 Z1P | A1bL4aL3B1G0 | 69.5 | 167 | 24.92 | 87.5 | 45.5 | 1800* | <5 | 55 | 25 | 71.5 | 169 | 25.03 | 81.7 | 45 | 150 | <5 | 2.5 | 2 |
| 26 Z1P | A1bL2B1G0p | 44.5 | 155 | 18.52 | 76.5 | 36.2 | 5183 | 44.5 | 52.5 | 24 | 48.5 | 155.5 | 20.06 | 88.2 | 46.2 | 1470 | 9.9 | 5 | 12 |
| 27 Z1P | A1bL4aL3B1G0 | 54 | 161 | 20.83 | 74.1 | 42.3 | 3957 | 23 | 52.5 | 35 | 58.6 | 161 | 22.61 | 79.7 | 47.8 | 126 | 16 | 5 | 3 |
| 28Z1P | A2bL4aL1B2G1 | 44.2 | 153 | 18.88 | 71.2 | 41.4 | 4456 | 8 | 55 | 22 | 51.9 | 155 | 21.6 | 71.9 | 40.6 | 159 | 19 | 5 | 0 |

IFX – infliximab, PCDAI - the pediatric Crohn’s disease activity index; SES-CD - the simplified endoscopic scale for Crohn’s disease;
