# Supplementary material for: Changes in the Intestinal Microbiota Are Seen Following Treatment with Infliximab in Children with Crohn’s Disease
Source: J Clin Med. 2020 Mar 4;9(3):687. doi: 10.3390/jcm9030687 (PMC7141282; doi:10.3390/jcm9030687)
Supplement: Supplementary file 1 [file jcm-09-00687-s001.zip › Supplement5.docx]

**Supplement Material 5. Short annotation of 32 bacteria observed only in CD patients in the context of human health.**

| **Taxonomy** | **what is known about them in the context of human health** | **Reference** | **[PMID]** |
| --- | --- | --- | --- |
| *Corynebacterium durum* | isolated from respiratory tract; dental pathogenesis | (Riegel et al. 1997; Rassoulian Barrett et al. 2001) | [9336915](https://www.ncbi.nlm.nih.gov/pubmed/9336915) [11230408](https://www.ncbi.nlm.nih.gov/pubmed/11230408) |
| Unassinged from family *Propionibacteriaceae* | a new name 'C*utibacterium*' - skin microbiota member; acne; | (Ramasamy et al. 2019) | [31342510](https://www.ncbi.nlm.nih.gov/pubmed/31342510) |
| *Propionibacterium acnes* |  |  |  |
| *Bifidobacterium breve* | new borne gut and human milk microbiota member; probiotics | (Kordy et al. 2020; Maldonado et al. 2019) | [31990909](https://www.ncbi.nlm.nih.gov/pubmed/31990909) [31630683](https://www.ncbi.nlm.nih.gov/pubmed/31630683) |
| Unclassified from genus *Atopobium* | vaginal and gut microbiota member and bacterial vaginosis | (Mendling et al. 2019; Thorasin, Hoyles, and McCartney 2015) | [30953190](https://www.ncbi.nlm.nih.gov/pubmed/30953190) [25533445](https://www.ncbi.nlm.nih.gov/pubmed/25533445) |
| *Parabacteroides distasonis* | member of human gut microbiota; decreasing weight gain, hyperglycemia, and hepatic steatosis | (Wang et al. 2019) | [30605678](https://www.ncbi.nlm.nih.gov/pubmed/30605678) |
| Unclassified from genus *Staphylococcus* | member of human skin microbiota; some serious infection including sepsis | (Otto 2014; Cao et al. 2020) | [24222452](https://www.ncbi.nlm.nih.gov/pubmed/24222452) [32082571](https://www.ncbi.nlm.nih.gov/pubmed/32082571) |
| Unclassified from family *Gemellaceae* | the mucous membranes of humans in the oral cavity and upper digestive tract - pulmonary exacerbations of cystic fibrosis; endocarditis; valvular disease | (Carmody et al. 2013; Mosquera et al. 2000) | [23802813](https://www.ncbi.nlm.nih.gov/pubmed/23802813) [11168056](https://www.ncbi.nlm.nih.gov/pubmed/11168056) |
| Unclassified from genus *Abiotrophia* | some time endocarditis | (Sharaf and Shaikh 2005; Bhat et al. 2014) | [16341303](https://www.ncbi.nlm.nih.gov/pubmed/16341303) [23682722](https://www.ncbi.nlm.nih.gov/pubmed/23682722) |
| Unclassified from genus *Enterococcus* | member of gut microbiota - endocarditis; sepsis; intra-abdominal abscesses; prostatitis; urinary tract infections | (Amyes 2007; IKE 2017) | [17659211](https://www.ncbi.nlm.nih.gov/pubmed/17659211) [28659548](https://www.ncbi.nlm.nih.gov/pubmed/17659211) |
| Unclassified from family *Lactobacillaceae* | member of gut, vagina and oral cavity microbiota - caries inhibition; endocarditis; alleviation of the symptom of irritable bowel syndrome; probiotics | (Ortiz-Lucas et al. 2013; Moosavi and Zuckerman 2019; Groga-Bada et al. 2018) | [23548007](https://www.ncbi.nlm.nih.gov/pubmed/23548007) [31929930](https://www.ncbi.nlm.nih.gov/pubmed/31929930) [30254678](https://www.ncbi.nlm.nih.gov/pubmed/30254678) |
| *Lactobacillus salivarius* |  |  |  |
| Unclassified from genus *Pediococcus* | member of gut microbiota; bacteriocin producer | (Porto et al. 2017; Stedman et al. 2020) | [28284993](https://www.ncbi.nlm.nih.gov/pubmed/28284993) [32026493](https://www.ncbi.nlm.nih.gov/pubmed/32026493) |
| *Streptococcus sobrinus* | the oral cavity microbiota member; enhances the formation of caries; dental abscesses | (Wu et al. 2003; Scalioni et al. 2017) | [12925830](https://www.ncbi.nlm.nih.gov/pubmed/12925830) [28678943](https://www.ncbi.nlm.nih.gov/pubmed/28678943) |
| *Clostridium paraputrificum* | anaerobic bacteremia; colonic necrosis | (Shinha and Hadi 2015; Kwon et al. 2018) | [25692054](https://www.ncbi.nlm.nih.gov/pubmed/25692054) [29599902](https://www.ncbi.nlm.nih.gov/pubmed/29599902) |
| *Clostridium perfringens* | gas gangrene | (Nagahama, Takehara, and Rood 2018; Gomes, Hoffmann, and Mota 2018) | [31350831](https://www.ncbi.nlm.nih.gov/pubmed/31350831) |
| Unclassified from genus *Clostridium* | member of gut microbiota |  | [29667480](https://www.ncbi.nlm.nih.gov/pubmed/29667480) |
| *Clostridium hathewayi* | rarely bacteremia and surgical site infection | (Elsayed and Zhang 2004; Dababneh et al. 2014) | [15550205](https://www.ncbi.nlm.nih.gov/pubmed/15550205) [24596408](https://www.ncbi.nlm.nih.gov/pubmed/24596408) |
| Unclassified from genus *Epulopiscium* | symbiotic fish related bacteria - no data on human infections | (Angert, Brooks, and Pace 1996; Miyake, Ngugi, and Stingl 2016) | [8631724](https://www.ncbi.nlm.nih.gov/pubmed/8631724) [27014209](https://www.ncbi.nlm.nih.gov/pubmed/27014209) |
| Unclassified from genus *Peptococcus* | member of gut and subgingival microbiota; Periodontitis | (Shi et al. 2018) | [29765908](https://www.ncbi.nlm.nih.gov/pubmed/29765908) |
| Unclassified from family *Peptostreptococcaceae* | member of the lower reproductive tract of women - pelvic inflammatory disease and bacterial vaginosis. Colorectal carcinogenesis | (Senok et al. 2009; Long et al. 2019) | [19821358](https://www.ncbi.nlm.nih.gov/pubmed/19821358) [31501538](https://www.ncbi.nlm.nih.gov/pubmed/31501538) |
| *Peptostreptococcus anaerobius* |  |  |  |
| Unclassified from genus *Anaerotruncus* | member of vaginal and gut microbiota; obese; endometrial cancer | (Togo et al. 2019; Walther-António et al. 2016) | [30891246](https://www.ncbi.nlm.nih.gov/pubmed/30891246) [27884207](https://www.ncbi.nlm.nih.gov/pubmed/27884207) |
| Unclassified from genus *Veillonella* | member of the oral cavity and gut microbiota; osteomyelitis and endocarditis | (Mashima et al. 2017; Houston, Taylor, and Rennie 1997; Baker and Allyn 2017) | [28934367](https://www.ncbi.nlm.nih.gov/pubmed/28934367) [9142818](https://www.ncbi.nlm.nih.gov/pubmed/9142818) [29026635](https://www.ncbi.nlm.nih.gov/pubmed/29026635) |
| Veillonella dispar |  |  |  |
| *Clostridium spiroforme* | enteric diseases in humans and/or animals | (Uzal et al. 2018) | [29883627](https://www.ncbi.nlm.nih.gov/pubmed/29883627) |
| *Coprobacillus cateniformis* | member of gut microbiota; irritable bowel syndrome | (Kageyama and Benno 2000; Lyra et al. 2009) | [10711596](https://www.ncbi.nlm.nih.gov/pubmed/10711596) [20014457](https://www.ncbi.nlm.nih.gov/pubmed/20014457) |
| Unclassified from family Bradyrhizobiaceae | plant-associated bacteria; Cord colitis syndrome | (Gorkiewicz, Trajanoski, and Högenauer 2013) | [24195569](https://www.ncbi.nlm.nih.gov/pubmed/24195569) |
| Unclassified from genus Eikenella | member of the oral cavity and upper respiratory tract microbiota - endocarditis | (Sharara et al. 2016) | [27124204](https://www.ncbi.nlm.nih.gov/pubmed/27124204) |
| Unclassified from genus Klebsiella | pneumonia; sepsis | (Estell et al. 2016; Delatour et al. 2018) | [26492860](https://www.ncbi.nlm.nih.gov/pubmed/26492860) [29033357](https://www.ncbi.nlm.nih.gov/pubmed/29033357) |
| Morganella morganii | postoperative and other nosocomial infections; urinary tract infections; sepsis | (Erlanger et al. 2019; Mbelle et al. 2020) | [28919283](https://www.ncbi.nlm.nih.gov/pubmed/28919283) [31630429](https://www.ncbi.nlm.nih.gov/pubmed/31630429) |
| Unclassified from order CW040 | probably oral cavity microbiota member - infection in the course of cystic fibrosis | (Bor et al. 2019; Renwick et al. 2014) | [30894042](https://www.ncbi.nlm.nih.gov/pubmed/30894042) [25526264](https://www.ncbi.nlm.nih.gov/pubmed/25526264) |

**Bibliography:**

Amyes, Sebastian G.B. 2007. “Enterococci and Streptococci.” *International Journal of Antimicrobial Agents* 29 (SUPPL. 3). https://doi.org/10.1016/S0924-8579(07)72177-5.

Angert, Esther R., Austin E. Brooks, and Norman R. Pace. 1996. “Phylogenetic Analysis of Metabacterium Polyspora: Clues to the Evolutionary Origin of Daughter Cell Production in Epulopiscium Species, the Largest Bacteria.” *Journal of Bacteriology* 178 (5): 1451–56. https://doi.org/10.1128/jb.178.5.1451-1456.1996.

Baker, Sarah, and Rebecca Allyn. 2017. “Lytic Lesions: Looking Lethal but Leaving Room for a Simple Cure? A Case of Veillonella Spinal Osteomyelitis.” *JMM Case Reports* 4 (8). https://doi.org/10.1099/jmmcr.0.005108.

Bhat, Deepti P., Lakshmi Nagaraju, Basim I. Asmar, and Sanjeev Aggarwal. 2014. “Abiotrophia Endocarditis in Children with No Underlying Heart Disease: A Rare but a Virulent Organism.” *Congenital Heart Disease* 9 (4). https://doi.org/10.1111/chd.12095.

Bor, B., J. K. Bedree, W. Shi, J. S. McLean, and X. He. 2019. “Saccharibacteria (TM7) in the Human Oral Microbiome.” *Journal of Dental Research* 98 (5): 500–509. https://doi.org/10.1177/0022034519831671.

Cao, Yi, Alessander O Guimaraes, Melicent C Peck, Oleg Mayba, Felicia Ruffin, Kyu Hong, Montserrat Carrasco-Triguero, Vance G Fowler, Stacey A Maskarinec, and Carrie M Rosenberger. 2020. “Risk Stratification Biomarkers for Staphylococcus Aureus Bacteraemia.” *Clinical & Translational Immunology* 9 (2): e1110. https://doi.org/10.1002/cti2.1110.

Carmody, Lisa A., Jiangchao Zhao, Patrick D. Schloss, Joseph F. Petrosino, Susan Murray, Vincent B. Young, Jun Z. Li, and John J. LiPuma. 2013. “Changes in Cystic Fibrosis Airway Microbiota at Pulmonary Exacerbation.” *Annals of the American Thoracic Society* 10 (3): 179–87. https://doi.org/10.1513/AnnalsATS.201211-107OC.

Dababneh, Ala S., Avish Nagpal, Bharath Raj Varatharaj Palraj, and M. Rizwan Sohail. 2014. “Clostridium Hathewayi Bacteraemia and Surgical Site Infection after Uterine Myomectomy.” *BMJ Case Reports* 2014 (March). https://doi.org/10.1136/bcr-2013-009322.

Delatour, Camille, Nellie Chalvon, Nathalie Prieur, and Philippe Mateu. 2018. “A History of Community-Acquired Hypervirulent Klebsiella Pneumoniae Severe Sepsis.” *Anaesthesia Critical Care and Pain Medicine* 37 (3): 273–75. https://doi.org/10.1016/j.accpm.2017.09.003.

Elsayed, Sameer, and Kunyan Zhang. 2004. “Human Infection Caused by Clostridium Hatheawayi.” *Emerging Infectious Diseases* 10 (11): 1950–52. https://doi.org/10.3201/eid1011.040006.

Erlanger, David, Marc Victor Assous, Yonit Wiener-Well, Amos Moshe Yinnon, and Eli Ben-Chetrit. 2019. “Clinical Manifestations, Risk Factors and Prognosis of Patients with Morganella Morganii Sepsis.” *Journal of Microbiology, Immunology and Infection* 52 (3): 443–48. https://doi.org/10.1016/j.jmii.2017.08.010.

Estell, K. E., A. Young, T. Kozikowski, E. A. Swain, B. A. Byrne, C. M. Reilly, P. H. Kass, and M. Aleman. 2016. “Pneumonia Caused by Klebsiella Spp. in 46 Horses.” *Journal of Veterinary Internal Medicine* 30 (1): 314–21. https://doi.org/10.1111/jvim.13653.

Gomes, Aline Corado, Christian Hoffmann, and João Felipe Mota. 2018. “The Human Gut Microbiota: Metabolism and Perspective in Obesity.” *Gut Microbes* 9 (4): 308–25. https://doi.org/10.1080/19490976.2018.1465157.

Gorkiewicz, Gregor, Slave Trajanoski, and Christoph Högenauer. 2013. “Bradyrhizobium Enterica in Cord Colitis Syndrome.” *New England Journal of Medicine* 369 (19): 1866–67. https://doi.org/10.1056/NEJMc1311318.

Groga-Bada, Patrick, Iris I. Mueller, Federico Foschi, Meinrad Gawaz, and Christian Eick. 2018. “Mitral Valve Endocarditis Due to Lactobacillus.” *Case Reports in Medicine* 2018. https://doi.org/10.1155/2018/8613948.

Houston, S., D. Taylor, and R. Rennie. 1997. “Prosthetic Valve Endocarditis Due to Veillonella Dispar: Successful Medical Treatment Following Penicillin Desensitization.” *Clinical Infectious Diseases* 24 (5): 1013–14. https://doi.org/10.1093/clinids/24.5.1013.

IKE, Yasuyoshi. 2017. “Pathogenicity of Enterococci.” *Nippon Saikingaku Zasshi* 72 (2): 189–211. https://doi.org/10.3412/jsb.72.189.

Kageyama, Akiko, and Yoshimi Benno. 2000. “Coprobacillus Catenaformis Gen. Nov., Sp. Nov., a New Genus and Species Isolated from Human Feces.” *Microbiology and Immunology* 44 (1): 23–28. https://doi.org/10.1111/j.1348-0421.2000.tb01242.x.

Kordy, Kattayoun, Thaidra Gaufin, Martin Mwangi, Fan Li, Chiara Cerini, David J. Lee, Helty Adisetiyo, et al. 2020. “Contributions to Human Breast Milk Microbiome and Enteromammary Transfer of Bifidobacterium Breve.” *PLoS ONE* 15 (1). https://doi.org/10.1371/journal.pone.0219633.

Kwon, Yong K., Faiqa A. Cheema, Bejon T. Maneckshana, Caroline Rochon, and Patricia A. Sheiner. 2018. “Clostridium Paraputrificum Septicemia and Liver Abscess.” *World Journal of Hepatology* 10 (3): 388–95. https://doi.org/10.4254/wjh.v10.i3.388.

Long, Xiaohang, Chi Chun Wong, Li Tong, Eagle S H Chu, Chun Ho Szeto, Minne Y Y Go, Olabisi Oluwabukola Coker, et al. 2019. “Peptostreptococcus Anaerobius Promotes Colorectal Carcinogenesis and Modulates Tumour Immunity.” *Nature Microbiology* 4 (12): 2319–30. https://doi.org/10.1038/s41564-019-0541-3.

Lyra, Anna, Teemu Rinttilä, Janne Nikkilä, Lotta Krogius-Kurikka, Kajsa Kajander, Erja Malinen, Jaana Mättö, Laura Mäkelä, and Airi Palva. 2009. “Diarrhoea-Predominant Irritable Bowel Syndrome Distinguishable by 16S RRNA Gene Phylotype Quantification.” *World Journal of Gastroenterology* 15 (47): 5936–45. https://doi.org/10.3748/wjg.15.5936.

Maldonado, J., M. Gil-Campos, J. A. Maldonado-Lobón, M. R. Benavides, K. Flores-Rojas, R. Jaldo, I. Jiménez Del Barco, et al. 2019. “Evaluation of the Safety, Tolerance and Efficacy of 1-Year Consumption of Infant Formula Supplemented with Lactobacillus Fermentum CECT5716 Lc40 or Bifidobacterium Breve CECT7263: A Randomized Controlled Trial.” *BMC Pediatrics* 19 (1): 456頁、453頁、603頁. https://doi.org/10.1186/s12887-019-1753-7.

Mashima, Izumi, Citra F. Theodorea, Boonyanit Thaweboon, Sroisiri Thaweboon, Frank A. Scannapieco, and Futoshi Nakazawa. 2017. “Exploring the Salivary Microbiome of Children Stratified by the Oral Hygiene Index.” *PLoS ONE* 12 (9). https://doi.org/10.1371/journal.pone.0185274.

Mbelle, N., J. Osei Sekyere, C. Feldman, N. E. Maningi, L. Modipane, and S. Y. Essack. 2020. “Genomic Analysis of Two Drug-Resistant Clinical Morganella Morganii Strains Isolated from UTI Patients in Pretoria, South Africa.” *Letters in Applied Microbiology* 70 (1): 21–28. https://doi.org/10.1111/lam.13237.

Mendling, Werner, Ana Palmeira-de-Oliveira, Stephan Biber, and Valdas Prasauskas. 2019. “An Update on the Role of Atopobium Vaginae in Bacterial Vaginosis: What to Consider When Choosing a Treatment? A Mini Review.” *Archives of Gynecology and Obstetrics* 300 (1): 1–6. https://doi.org/10.1007/s00404-019-05142-8.

Miyake, Sou, David K Ngugi, and Ulrich Stingl. 2016. “Phylogenetic Diversity, Distribution, and Cophylogeny of Giant Bacteria (Epulopiscium) with Their Surgeonfish Hosts in the Red Sea.” *Frontiers in Microbiology* 7: 285. https://doi.org/10.3389/fmicb.2016.00285.

Moosavi, Mitchell, and Jonathan E. Zuckerman. 2019. “ Lactobacillus Endocarditis-Associated Glomerulonephritis Complicated by Anti-Coagulant Nephropathy and Renal Amyloidosis .” *Case Reports in Pathology* 2019: 1–3. https://doi.org/10.1155/2019/6198380.

Mosquera, J. D., M. Zabalza, M. Lantero, and J. R. Blanco. 2000. “Endocarditis Due to Gemella Haemolysans in a Patient with Hemochromatosis [6].” *Clinical Microbiology and Infection* 6 (10): 566–68. https://doi.org/10.1046/j.1469-0691.2000.00136.x.

Nagahama, Masahiro, Masaya Takehara, and Julian I Rood. 2018. “Histotoxic Clostridial Infections.” *Microbiology Spectrum* 6 (4). https://doi.org/10.1128/microbiolspec.GPP3-0024-2018.

Ortiz-Lucas, María, Aurelio Tobias, Pablo Saz, and Juan José Sebastián. 2013. “Efecto de Los Probióticos En Los Síntomas Del Síndrome Del Intestino Irritable: Un Meta-Análisis Actualizado.” *Revista Espanola de Enfermedades Digestivas* 105 (1): 19–36. https://doi.org/10.4321/S1130-01082013000100005.

Otto, Michael. 2014. “Staphylococcus Epidermidis Pathogenesis.” *Methods in Molecular Biology* 1106: 17–31. https://doi.org/10.1007/978-1-62703-736-5_2.

Porto, Maria Carolina W., Taís Mayumi Kuniyoshi, P. O.S. Azevedo, Michele Vitolo, and R. P.S. Oliveira. 2017. “Pediococcus Spp.: An Important Genus of Lactic Acid Bacteria and Pediocin Producers.” *Biotechnology Advances* 35 (3): 361–74. https://doi.org/10.1016/j.biotechadv.2017.03.004.

Ramasamy, S., E. Barnard, T. L. Dawson, and H. Li. 2019. “The Role of the Skin Microbiota in Acne Pathophysiology.” *British Journal of Dermatology* 181 (4): 691–99. https://doi.org/10.1111/bjd.18230.

Rassoulian Barrett, S. L., B. T. Cookson, L. C. Carlson, K. A. Bernard, and M. B. Coyle. 2001. “Diversity within Reference Strains of Corynebacterium Matruchotii Includes Corynebacterium Durum and a Novel Organism.” *Journal of Clinical Microbiology* 39 (3): 943–48. https://doi.org/10.1128/JCM.39.3.943-948.2001.

Renwick, Julie, Paul McNally, Bettina John, Todd DeSantis, Barry Linnane, and Philip Murphy. 2014. “The Microbial Community of the Cystic Fibrosis Airway Is Disrupted in Early Life.” *PLoS ONE* 9 (12). https://doi.org/10.1371/journal.pone.0109798.

Riegel, Philippe, Remy Heller, Gilles Prevost, François Jehl, and Henri Monteil. 1997. “Corynebacterium Durum Sp. Nov., from Human Clinical Specimens.” *International Journal of Systematic Bacteriology* 47 (4): 1107–11. https://doi.org/10.1099/00207713-47-4-1107.

Scalioni, Flávia, Camila Carrada, Fernanda Machado, Karina Devito, Luiz Cláudio Ribeiro, Dionéia Cesar, and Rosangela Ribeiro. 2017. “Salivary Density of Streptococcus Mutans and Streptococcus Sobrinus and Dental Caries in Children and Adolescents with down Syndrome.” *Journal of Applied Oral Science* 25 (3): 250–57. https://doi.org/10.1590/1678-7757-2016-0241.

Senok, Abiola C, Hans Verstraelen, Marleen Temmerman, and Giuseppe A Botta. 2009. “Probiotics for the Treatment of Bacterial Vaginosis.” *The Cochrane Database of Systematic Reviews*, no. 4 (October): CD006289. https://doi.org/10.1002/14651858.CD006289.pub2.

Sharaf, Mahmoud A, and Nasir Shaikh. 2005. “Abiotrophia Endocarditis: Case Report and Review of the Literature.” *The Canadian Journal of Cardiology* 21 (14): 1309–11. http://www.ncbi.nlm.nih.gov/pubmed/16341303.

Sharara, Sima L, Ralph Tayyar, Zeina A Kanafani, and Souha S Kanj. 2016. “HACEK Endocarditis: A Review.” *Expert Review of Anti-Infective Therapy* 14 (6): 539–45. https://doi.org/10.1080/14787210.2016.1184085.

Shi, Meng, Yiping Wei, Wenjie Hu, Yong Nie, Xiaolei Wu, and Ruifang Lu. 2018. “The Subgingival Microbiome of Periodontal Pockets with Different Probing Depths in Chronic and Aggressive Periodontitis: A Pilot Study.” *Frontiers in Cellular and Infection Microbiology* 8 (MAY). https://doi.org/10.3389/fcimb.2018.00124.

Shinha, Takashi, and Christiane Hadi. 2015. “Clostridium Paraputrificum Bacteremia Associated with Colonic Necrosis in a Patient with AIDS .” *Case Reports in Infectious Diseases* 2015: 1–3. https://doi.org/10.1155/2015/312919.

Stedman, Anna, Arnoud H M van Vliet, Mark A Chambers, and Jorge Gutierrez-Merino. 2020. “Gut Commensal Bacteria Show Beneficial Properties as Wildlife Probiotics.” *Annals of the New York Academy of Sciences*, February. https://doi.org/10.1111/nyas.14302.

Thorasin, Thanikan, Lesley Hoyles, and Anne L. McCartney. 2015. “Dynamics and Diversity of the ‘Atopobium Cluster’ in the Human Faecal Microbiota, and Phenotypic Characterization of ‘Atopobium Cluster’ Isolates.” *Microbiology (United Kingdom)* 161 (3): 565–79. https://doi.org/10.1099/mic.0.000016.

Togo, A. H., A. Diop, G. Dubourg, S. Khelaifia, M. Richez, N. Armstrong, M. Maraninchi, P. E. Fournier, D. Raoult, and M. Million. 2019. “Anaerotruncus Massiliensis Sp. Nov., a Succinate-Producing Bacterium Isolated from Human Stool from an Obese Patient after Bariatric Surgery.” *New Microbes and New Infections* 29. https://doi.org/10.1016/j.nmni.2019.01.004.

Uzal, Francisco A, Mauricio A Navarro, Jihong Li, John C Freedman, Archana Shrestha, and Bruce A McClane. 2018. “Comparative Pathogenesis of Enteric Clostridial Infections in Humans and Animals.” *Anaerobe* 53 (October): 11–20. https://doi.org/10.1016/j.anaerobe.2018.06.002.

Walther-António, Marina R.S., Jun Chen, Francesco Multinu, Alexis Hokenstad, Tammy J. Distad, E. Heidi Cheek, Gary L. Keeney, et al. 2016. “Potential Contribution of the Uterine Microbiome in the Development of Endometrial Cancer.” *Genome Medicine* 8 (1). https://doi.org/10.1186/s13073-016-0368-y.

Wang, Kai, Mingfang Liao, Nan Zhou, Li Bao, Ke Ma, Zhongyong Zheng, Yujing Wang, et al. 2019. “Parabacteroides Distasonis Alleviates Obesity and Metabolic Dysfunctions via Production of Succinate and Secondary Bile Acids.” *Cell Reports* 26 (1): 222-235.e5. https://doi.org/10.1016/j.celrep.2018.12.028.

Wu, Hongkun, Mingwen Fan, Xuedong Zhou, Anchun Mo, Zhuan Bian, Qi Zhang, and Zhi Chen. 2003. “Detection of Streptococcus Mutans and Streptococcus Sobrinus on the Permanent First Molars of the Mosuo People in China.” *Caries Research* 37 (5): 374–80. https://doi.org/10.1159/000072171.
